# Assessment of Physicochemical Parameters in Two Winegrapes Varieties after Foliar Application of ZnSO_4_ and ZnO

**DOI:** 10.3390/plants12071426

**Published:** 2023-03-23

**Authors:** Diana Daccak, Fernando C. Lidon, Ana Rita F. Coelho, Inês Carmo Luís, Ana Coelho Marques, Cláudia Campos Pessoa, Maria da Graça Brito, José Carlos Kullberg, José C. Ramalho, Maria José Silva, Ana Paula Rodrigues, Paula Scotti Campos, Isabel P. Pais, José N. Semedo, Maria Manuela Silva, Paulo Legoinha, Carlos Galhano, Manuela Simões, Maria Fernanda Pessoa, Fernando H. Reboredo

**Affiliations:** 1 Departamento de Ciências da Terra, Faculdade de Ciências e Tecnologia, Campus da Caparica, Universidade Nova de Lisboa, 2829-516 Caparica, Portugal; fjl@fct.unl.pt (F.C.L.); arf.coelho@campus.fct.unl.pt (A.R.F.C.); idc.rodrigues@campus.fct.unl.pt (I.C.L.); amc.marques@campus.fct.unl.pt (A.C.M.); c.pessoa@campus.fct.unl.pt (C.C.P.); mgb@fct.unl.pt (M.d.G.B.); jck@fct.unl.pt (J.C.K.); mma.silva@fct.unl.pt (M.M.S.); pal@fct.unl.pt (P.L.); acag@fct.unl.pt (C.G.); mmsr@fct.unl.pt (M.S.); mfgp@fct.unl.pt (M.F.P.); fhr@fct.unl.pt (F.H.R.); 2 Centro de Investigação de Geobiociências, Geoengenharias e Geotecnologias (GeoBioTec), Faculdade de Ciências e Tecnologia, Campus da Caparica, Universidade Nova de Lisboa, 2829-516 Caparica, Portugal; cochichor@mail.telepac.pt (J.C.R.); mjsilva@isa.ulisboa.pt (M.J.S.); paula.scotti@iniav.pt (P.S.C.); isabel.pais@iniav.pt (I.P.P.); jose.semedo@iniav.pt (J.N.S.); 3 Plant Stress & Biodiversity Lab, Centro de Estudos Florestais (CEF), Laboratório Associado TERRA, Instituto Superior Agronomia (ISA), Universidade de Lisboa (ULisboa), Quinta do Marquês, Avenida da República, 2784-505 Oeiras, Portugal; anadr@isa.ulisboa.pt; 4 Plant Stress & Biodiversity Lab, Centro de Estudos Florestais (CEF), Laboratório Associado TERRA, Instituto Superior Agronomia (ISA), Universidade de Lisboa (ULisboa), Tapada da Ajuda, 1349-017 Lisboa, Portugal; 5 Instituto Nacional de Investigação Agrária e Veterinária, I.P. (INIAV), Quinta do Marquês, Avenida da República, 2780-157 Oeiras, Portugal

**Keywords:** Castelão, Syrah, winegrapes, winemaking, zinc biofortification

## Abstract

One-third of the world’s population is suffering from “hidden hunger” due to micronutrient deficiency. Zinc is acquired through diet, leading its deficiency to the development of disorders such as retarded growth, anorexia, infections, and hypogeusia. Accordingly, this study aimed to develop an agronomic workflow for Zn biofortification on two red winegrapes varieties (cv. Castelão and Syrah) and determine the physicochemical implications for winemaking. Both varieties produced in Setúbal (Portugal) were submitted to four foliar applications of ZnSO_4_ or ZnO (900 and 1350 g ha^−1^, respectively), during the production cycle. At harvest, Zn biofortification reached a 4.3- and 2.3-fold increase with ZnO 1350 g ha^−1^ in Castelão and Syrah, respectively (although, with ZnSO_4_ 1350 g ha^−1^ both varieties revealed an increase in Zn concentration). On a physiological basis, lower values of NDVI were found in the biofortified grapes, although not reflected in photosynthetic parameters with cv. Syrah shows even a potential benefit with the use of Zn fertilizers. Regarding physical and chemical parameters (density, total soluble solids, dry weight, and color), relative to the control no significant changes in both varieties were observed, being suitable for winemaking. It was concluded that ZnSO_4_ and ZnO foliar fertilization efficiently increased Zn concentration on both varieties without a negative impact on quality, but cv. Castelão showed a better index of Zn biofortification and pointed to a potentially higher quality for winemaking.

## 1. Introduction

Micronutrient deficiency also known as “hidden hunger” is considered a public health problem, affecting about two billion people (ca., one-third of the world’s population) worldwide [1,2]. Globally, 800 million people are undernourished and 1.5–2 billion people have chronic micronutrient deficiencies due to the lack of calcium, iodine, iron, selenium, zinc, and vitamins such as folate and vitamin A [2]. In this context, Zn deficiency is one of the most common, leading to adverse effects in several organ systems (e.g., brain development and cognition) and consequently leading to diseases and disorders, namely blindness, decrease in IQ level, infections and mortality during pregnancy, retarded growth, anorexia and hypogeusia in children [3,4,5,6,7]. In addition, Zn below the required level is related to the worsening of the development of neurological, autoimmune, and cardiovascular diseases and diabetes mellitus [8]. To meet the organism’s needs, the Zn amount of 10–15 mg in adults is considered the recommended daily intake, with a higher requirement in pregnancy and lactation. Through diet, this micronutrient is found in several foods, including meat, legumes, oysters, crab, fish, nuts, grains cereals, and others [7,9,10].

As a strategy to mitigate Zn deficiency, agronomic biofortification has been applied to several crops. This approach leads to an increase in nutrient concentration in edible parts of plants, through agronomic practices [11,12,13,14]. Fertilization can be performed through soil and/or foliar application, with this last avoiding fixation and immobilization of nutrients in soils at toxic levels to plants [15,16,17]. Indeed, relative to soil application, foliar spraying allows higher efficiency and translocation of nutrients during the productive cycle [15,16], as was observed for chickpeas [18]. Additionally, biofortification through foliar spraying already showed positive effects in rice and wheat regarding the yield, quality, and Zn concentration in grains [19]. Nevertheless, despite the greater efficiency of foliar application, the absorption of nutrients is dependent on the upper and lower leaf surface thickness of the cuticle of each specie, as well as the number of pores, distribution of trichomes and stomata of the leaf surface [20,21]. In addition, Zn translocation (as Zn^2+^ or bounded to organic acids) is dependent on the remobilization through xylem or phloem (i.e., Zn mobility is higher in phloem compared to xylem) to the growing tissues [22,23,24]. Transport of Zn involves proteins namely, the Zn and Fe permease family (ZIP family), the heavy metal ATPase (HMA), and the metal tolerant proteins (MTP), as well as non-selective cation channels (e.g., Ca channels in plasmatic membranes) [24,25,26].

Zinc occurs in the six enzyme classes (Enzyme Commission Number, EC 1–6: oxidoreductases, transferases, hydrolases, lyases, isomerases, ligases), with numerous binding sites in proteins, lipids, and nucleic acid molecules [3,27,28,29]. The zinc finger domain is the largest Zn-binding protein class, controlling the proliferation and differentiation of cells (i.e., with effects in DNA/RNA-binding, site-specific modifications, regulation of chromatin structure, protein interactions, and others) [3,28,29,30]. Following a physiological perspective, in plants, Zn is involved in several functions, with catalytic (present in about 300 proteins such as carbonic anhydrase), structural (e.g., protein kinases and alcohol dehydrogenases), and regulatory activities [31,32]. Indeed, it affects the photosynthetic process (development of chloroplasts and repair process of the photosystem II involving the D1 protein and SPP peptidase) [33], auxin metabolism, proteosynthesis and metabolism of carbohydrates, lipids, and nucleic acids, expression and regulation of genes, cell membrane integrity, control of oxy radicals and several others physiological functions [34,35,36,37,38,39]. Considering the physiological implications of Zn to optimal growth, most crops require 15–20 mg kg^−1^_dw_ [40]. Insufficient levels of Zn can trigger extensive oxidative damage to membrane lipids, proteins, chlorophyll, and nucleic acids due to the generation of reactive oxygen species (ROS) [41,42]. Moreover, as mentioned, Zn is involved in the activity of carbonic anhydrase, whose imbalance can impair the transfer of CO_2_/HCO_3_ in the leaf, necessary for the photosynthetic fixation of CO_2_ [43]. These biochemical changes, in Zn severe deficiency conditions, lead to symptoms, such as stunting of plants, root apex necrosis, chlorosis and necrotic spots on leaves, malformed leaves and inward curling of leaf lamina [44,45,46,47]. Nevertheless, negative effects can also occur when the Zn threshold of toxicity is surpassed (300–400 mg kg^−1^_dw_) [48]. Zinc toxicity can also have an effect on oxidative damage, chlorosis in the younger leaves and if persistent, extends to the older leaves, inhibition of photosystems I and II (reversibly if not under constant stress), and a decrease in plant growth [48,49].

The aim of this study was the development of an itinerary for Zn biofortification of two red grape varieties (Castelão and Syrah) through foliar application of ZnSO_4_ and ZnO, considering the potential modulation of physiological parameters mediated by these fertilizers during the production cycle of the vines. Additionally, the index of Zn accumulation and the physicochemical characteristics were assessed. This study will provide us with Zn agronomic biofortification data of fruit in natural climatic conditions, associated with the global wine market by developing a potential new product with more health benefits.

## 2. Results

### 2.1. Irrigation Water

**Figure 1:**
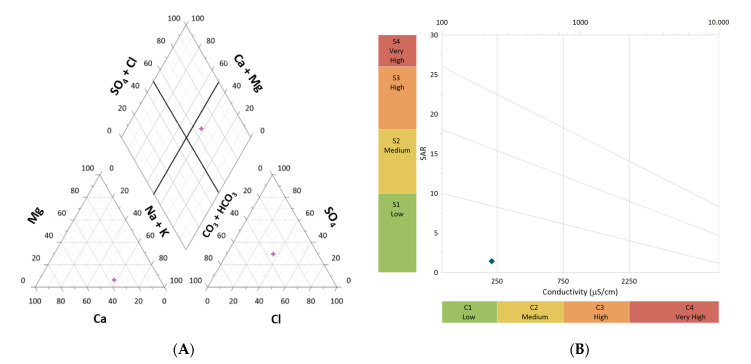
Physicochemical characterization of irrigation water in the vineyard of Syrah. Projection of water sample with (**A**) Ternary Piper diagram and (**B**) Wilcox diagram.

Water management in vineyards is essential to control vegetative growth and grape quality [50]. Thus, the irrigation water of the experimental field with Syrah was analyzed, considering the physicochemical properties. This water was from an underground origin, being found a pH of 6.2, with low salinity (concentration of salts evaluated, in terms of electrical conductivity (EC), between 100–250 μS/cm, at 20 °C) (Figure 1). The chemical composition of water plotted by the piper diagram is Na-SO_4_-Cl type (sodium–sulfate–chloride type), belonging to class C1S1 and with a sodium adsorption index (SAR) 1.4 (Figure 1).

### 2.2. Field Morphology and Vigor of the Vine

**Figure 2:**
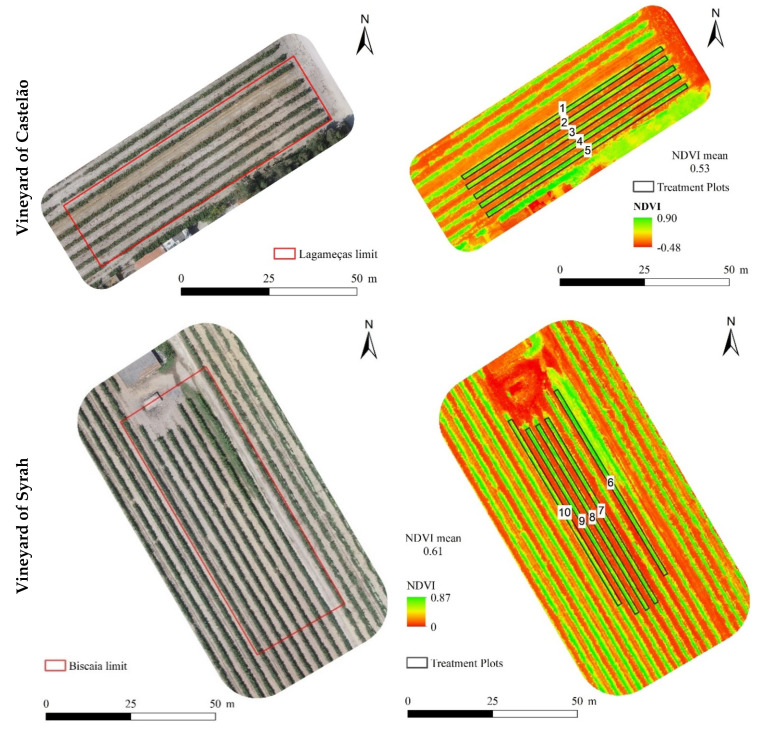
Orthophoto maps and normalized difference vegetation index (NDVI) of the vineyards of Castelão and Syrah. Information collected after the 4th foliar application of ZnSO_4_ or ZnO on 22 August 2019. Castelão: 1—control; 2—ZnO (900 g ha^−1^); 3—ZnO (1350 g ha^−1^); 4—ZnSO_4_ (900 g ha^−1^) and 5—ZnSO_4_ (900 g ha^−1^). Syrah: 6—control; 7—ZnO (900 g ha^−1^); 8—ZnO (1350 g ha^−1^); 9—ZnSO_4_ (900 g ha^−1^); 10—ZnSO_4_ (900 g ha^−1^).

After the fourth foliar application, the normalized difference vegetation index (NDVI) of both Castelão and Syrah vineyards revealed a higher vigor in the vines without foliar spraying with Zn chemical forms (although the vigor of the vines was similar with a predominance of green areas) (Figure 2; Table 1). Comparing the ZnSO_4_ and ZnO foliar applications, a better NDVI response was found with 900 g ha^−1^ of ZnSO_4_ in both varieties (revealing a value of 0.54 and 0.65 for Castelão and Syrah, respectively) (Figure 2). Additionally, Castelão showed a lower vigor (i.e., a range between 0.52–0.56) considering the mean vineyard values found with Syrah (i.e., a range between 0.55–0.66).

### 2.3. Leaf Gas Exchange Parameters

Net photosynthesis (Pn), stomatal conductance to water vapor (gs), transpiration (E), and leaf instantaneous water use efficiency (iWUE) of both vineyards Castelão and Syrah were monitored during the production cycle, to determine the potential physiological effects of Zn leaf spraying. After the fourth foliar spraying (21 August), significant differences could not be found for cv. Castelão and Syrah (i.e., except in Pn and E parameter for Syrah) (Table 2). Regarding Pn, Syrah sprayed with Zn chemical forms, revealed significantly higher values (i.e., except ZnO 900 g ha^−1^), whereas relative to the control, only E showed the highest concentration with ZnSO_4_ (1350 g ha^−1^) (Table 2). Moreover, the iWUE did not reveal a negative impact. In general, the Zn foliar spraying at all concentrations (900 and 1350 g ha^−1^ of ZnO and ZnSO_4_) maintained the performance of the photosynthetic machinery, even showing potential positive effects reflected in the Pn of Syrah (which may be related to the role of Zn in photosynthesis, namely in the enzyme carbonic anhydrase essential for the transport of carbon dioxide) [51].

### 2.4. Zn Concentration in the Leaf

**Figure 3:**
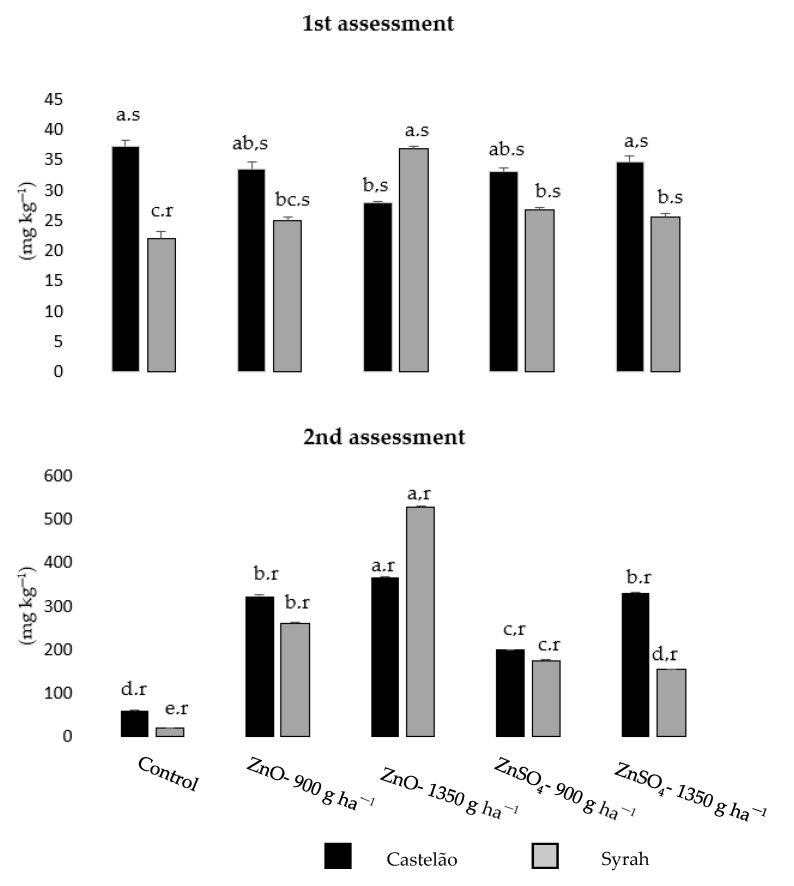
Average ± S.E. (*n* = 3) of Zn concentration in leaves of *Vitis vinifera,* varieties Castelão and Syrah, without applications and after the 4th foliar application of ZnSO_4_ or ZnO on 25 May (1st assessment) and 24 July 2019 (2nd assessment). Letters a, b, c, d, e indicate significant differences within the same column and variety, whereas letters r, s refer to significant differences in each variety between assessment dates for the same treatment (statistical analysis using the two-way ANOVA test, *p* < 0.05).

Before foliar spraying with ZnO or ZnSO_4_, Zn content in the leaves was similar among all treatments for Castelão (with ZnO 1350 g ha^−1^ showing a lower value), but some fluctuations were found in Syrah (with a minimum value in the control and maximum in ZnO 1350 g ha^−1^) (Figure 3). After the fourth foliar application of both Zn chemical forms, the Zn amount significantly increased in leaves of both varieties in all concentrations (with ZnO 1350 g ha^−1^ leading to the highest Zn concentration) (Figure 3). Between 25 May and 24 July, increased indexes of Zn were found for Castelão and Syrah, (relative to the leaves of the control, between 6.0–13.2- and 6.0–14.4-fold increase, respectively) (Figure 3).

### 2.5. Zn Concentration in the Grapes at Harvest

At harvest, ZnO and ZnSO_4_ sprayed grapes presented a higher Zn concentration compared to the control in both varieties (Table 3). The maximum increase was observed with 1350 g ha^−1^ of ZnO for both red grapes varieties (relative to control grapes, a 4.3 and 2.3-fold increase was found in Castelão and Syrah, respectively) (Table 3). In Zn-treated Castelão with 1350 g ha^−1^ of ZnSO_4_, a significant increase regarding the control grapes was also found (Table 3). Comparing both varieties, a higher Zn content was found in Syrah, observed significant differences for treatments 900 g ha^−1^ of ZnO and ZnSO_4_ and 1350 g ha^−1^ of ZnSO_4_ (Table 3).

### 2.6. Morphometric and Colorimetric Parameters of Grapes at Harvest

Still, at harvest, it was observed the absence of significant variations for the grapes of each variety in dry weight, density, and total soluble solids (TSS) (relative to control grapes, except 1350 g ha^−1^ of ZnO in Syrah and 900 g ha^−1^ of ZnSO_4_ in Castelão for total soluble solids) (Table 4). In Castelão and Syrah, the deviation of values found in dry weight ranged between 24.7–27.9% and 24.0–26.4%, respectively (yet significant differences were not detected between the two red grape varieties) (Table 4). As for the density and total soluble solids, in Castelão the values varied between 1274–1487 g cm^−3^ and 20.8–26.3 °Brix, whereas for Syrah 1049–1143 g cm^−3^ and 18.7–24.3 °Brix were quantified (Table 4). A comparative analysis between both varieties revealed differences in density and total soluble solids. Two treatments (control and ZnO 900 g ha^−1^) of Castelão were significantly higher than Syrah (Table 4).

Concerning the colorimetric analysis, the absence of significant variations was observed in L, a*, and b* parameters for both varieties (Table 5). Between Castelão and Syrah, some variations in a* and b* were found (mainly with the application of 1350 g ha^−1^ of both ZnO and ZnSO_4_) (Table 5). In addition, comparing both varieties, the L parameter showed a significantly lower value in Castelão (i.e., ZnO 1350 g ha^−1^).

## 3. Discussion

As the vines’ growth and quality depend on edaphoclimatic conditions, namely variety and viticultural management [52,53], two red winegrapes varieties of *Vitis vinifera*, in different vineyards of Setubal/Portugal, were used as experimental field trials for Zn biofortification. As water management through irrigation affects grape composition and wine quality [54], the production cycle of Syrah and Castelão varieties remained under different conditions (i.e., with and without irrigation, respectively). Moreover, both vineyards were situated in the same region and therefore with similar climatic influence (with year temperatures varying between 17.9–22.9 °C), which is considered close to the normal range for grapevine development (i.e., 12–22 °C) [55]. Regarding the Syrah vineyard submitted to irrigation, the water quality showed no restriction for agricultural use and is suitable in most soils without danger of salinization [56], as observed with the electric conductivity (i.e., 100–250 μS cm^−1^) and SAR value (i.e., 1.4) (Figure 1). Considering the response of Castelão and Syrah to Zn foliar spraying of both ZnSO_4_ and ZnO, the images where the normalized difference vegetation indices (NDVI) were calculated, are usually used to monitor the amount of photosynthetically active biomass, and also changes in crop states (i.e., diseases, environmental stress, water status, and others) [57,58,59]. As revealed by data of image acquisition found in both varieties (Figure 2; Table 1), NDVI values tended to decrease near the harvest period and with higher anthocyanin concentration (i.e., anthocyanin concentration increases at the maximum maturation point) [60,61,62]. Indeed, in this context, the red winegrape variety Cabernet Sauvignon also showed this negative relationship of NDVI with anthocyanin, with a high-vigor vine showing lower levels of anthocyanins [63]. Moreover, vines subjected to Zn foliar applications on both varieties Syrah and Castelão, presented a lower vigor compared to control vines (Figure 2; Table 1), being lower values also correlated with the best quality for winemaking [64]. Additionally, as canopy reflectance is dependent on leaf area index [62], these lower values of NDVI after Zn biofortification, indicated a higher proportion of fruits (thus, a higher yield) compared to leaf density. Complementarily, the photosynthetic parameters of both Castelão and Syrah varieties showed the absence of a negative impact (at the same stage of the images acquired for NDVI calculation), further supporting the maintenance of photoassimilates production (Table 2). Moreover, data acquired in Syrah showed a potential positive effect of Zn biofortification, namely with Pn (Table 2), which implicates additional chlorophyll synthesis and carbonic anhydrase activity, facilitating CO_2_ diffusion in the chloroplast [65]. In fact, it has been reported that Zn application improves photochemical reactions occurring in the thylakoid membrane, electron transport through PSII and increases the photosynthetic rate and chlorophyll content, leading to a higher fruit yield and quality, as previously observed for kinnow mandarin, sweet orange, and grapes [66].

Zinc has a high phloem mobility in the vine, being prone to the concentrations increase in plant organs [67,68]. Indeed, in Castelão and Syrah grapes, after the fourth Zn foliar spraying with ZnSO_4_ and ZnO, the leaves with a higher Zn content led to their higher accumulation in the fruit (Figure 3; Table 3). Zinc sulfate is the commonly used source of foliar application of Zn because of its high water solubility, yet other sources such as Zn-EDTA and ZnO are also being used, in spite of lower solubilities [69,70,71]. Moreover, relative to ZnO (which leads to a higher Zn supply when applied through the soil), similar bioavailability was observed in plants with foliar spraying with ZnSO_4_ [71]. Still, our data showed a similar response for Castelão and Syrah grapes with a higher efficiency with ZnO at 1350 g ha^−1^ (Table 3), suggesting a higher rate of translocation via the phloem to other tissues [72,73,74,75]. In addition, the use of ZnO for Zn biofortification can be advantageous in avoiding the threshold of toxicity, since it is gradually absorbed (with the opposite occurring with ZnSO_4_ which is rapidly acquired and can more easily lead to signs of toxicity) [76]. The differences in the Zn source efficiency can further be related to the different pathways that these chemical sources can follow, namely in the framework of hydrophobic and hydrophilic compounds, dissolution–diffusion processes, and through damaged cuticle tissue, epidermal structures such as the stomata, trichomes, and specialized epidermal cells, respectively [70].

At harvest, after Zn biofortification with both fertilizers, the absence of negative impacts on the quality parameters such as dry weight, density, °Brix, and color was found (Table 4). Data for the varieties of both red mature grapes have been reported to be optimal for °Brix within the range 13.7 to 31.5 [77] (i.e., a range of 18.7 to 26.3° in this study) and 70 to 85% for water content in winegrapes varieties [78] (i.e., a range of 72.1 to 76 in this study) (Table 4). Moreover, for greater quality wine, values between 22 and 28 °Brix are more advantageous [78,79]. Moreover, it is known that higher values of total soluble solids lead to a higher grape density [80]. Accordingly, our data corroborate this assumption in the grapes of Castelão, as they displayed higher density, the concentration of total soluble solids, and amount of dry matter (due to a higher compounds concentration such as: polyphenols and anthocyanins) linked to a lower water content (Table 4). Moreover, with Syrah the opposite occurred, evidencing the dilution effect with a higher water amount but lower sugar levels, as supported by [81]. These results were also observed in other winegrapes varieties, namely with Merlot and Cabernet Sauvignon compared to Garnacha and Tempranillo (with the largest berry size), where the smaller grapes were related to a more intense color and a higher number of anthocyanins and proanthocyanidins [82]. Notably, the NDVI results acquired corroborate these findings, with grapes of Castelão showing lower values relative to these of Syrah, which is explained by the higher concentration of anthocyanins (Figure 2; Table 4). It was also pointed out that in the Merlot Hamburg winegrape variety, densities superior to 1088 kg/m^3^ could have nutraceutical and sensory advantages [80], being these conditions were observed in Castelão and also in some cases of Syrah (Table 4). Nevertheless, colorimetric parameters did not show significative differences with Zn biofortification (Table 5), being similar to the values mentioned by [83] where L* varied between 17.74 to 60.27, a* from −17.19 to 18.11 and b* from −0.77 to 31.84. Additionally, it is well known that color is directly related to the concentration of anthocyanins, with the highest concentration indicating darker wines, as observed in previous studies [84,85]. In this respect, it was found that under irrigation lower values of anthocyanins were observed [86], a pattern also found for grapes of Syrah, as it appears to have a lower concentration relative to Castelão without irrigation (Table 5).

## 4. Materials and Methods

### 4.1. Experimental Plots and Treatments Applied

Two red grape varieties of *Vitis vinifera* L. (Castelão and Syrah, without and with irrigation, respectively), were used as a case study for Zn agronomic biofortification with ZnSO_4_ (AGROZAP) or ZnO (VITTIA) at concentrations of 0, 900 and 1350 g ha^−1^ (i.e., after flowering four foliar applications were applied during the production cycle between 29 June and 19 July 2019, being the control sprayed with water). Vineyard fields were located in Setúbal, Portugal with GPS coordinates 38°36′00.01376123″ N; 8°48′18.66998178″ W and 38°35′20.84975562″ N; 8°51′43.39046267″ W (for Castelão and Syrah varieties, respectively). Harvest was performed on 14 and 25 September of 2019 for Castelão and Syrah, respectively. Between 29 June (1st foliar application) and 25 September (latest harvest), maximum and minimum mean temperatures ranged between 22.7 and 17.9 °C, respectively. The air humidity revealed its maximum and minimum with values of 99% and 17%, and the total precipitation accumulated was 18.28 mm (with a daily maximum of 12.95 mm) which according to [87] this year is considered a dry summer.

### 4.2. Irrigation Water Analysis

Water quality of the vineyards was determined considering physical (pH, temperature, and electrical conductivity) and chemical (bicarbonate (HCO_3_^−^), sulfate (SO_4_^2−^), chloride (Cl^−^), sodium (Na^+^), calcium (Ca^2+^), magnesium (Mg^2+^), potassium (K^+^) and phosphate (PO_4_^3−^)) parameters. Electrical conductivity (EC) and pH were acquired using a Consort multiparameter analyzer (C 6030) and SP21 (pH) and SK20 T (CE) electrodes. Calcium, Na, K, and Mg ions were quantified using a chromatograph (Model 761 Compact IC, Metrohom, Herisau, Switzerland), equipped with column and pre-column (Metrosep cation 1–2, 6.1010.000), using an eluent mixture (4 mM tartaric acid/1 mM dipicolinic acid) at a flow rate of 1 mL/minute and a sample injection of 10 μL. Alkalinity/bicarbonate was determined with titration, in 100 mL of water samples, using 0.1 N hydrochloric acid as titrant, in the presence of 0.1% methyl orange [88]. Chloride, sulfate, nitrate, and phosphate ions were quantified through photometry (Spectroquant NOVA 60, Merck, Darmstadt, Germany), using specific kits (1.14897, 1.14779, 1.14773, and 1.14842). Water classification in the soils of both vineyards considered dominant ions [89]. Sodium adsorption index was determined and related to the electrical conductivity, in classes C and S [90]. Data acquired was projected in a piper and Wilcox diagram using Grapher software (version 16.3.410).

### 4.3. Field Morphology and Vigor of the Vine

Using a UAV (unmanned aerial vehicle) with altimetric measurement sensors and synchronized by GPS, images of both fields were acquired and processed in ArcGIS Pro (after the 4th foliar application of ZnSO_4_ or ZnO). To monitor the physiological response to Zn biofortification the orthophoto maps and NDVI index (normalized difference vegetation index) were obtained, according to [90,91].

### 4.4. Leaf Gas Exchange Parameters

Leaf gas exchange parameters were determined using 4–6 leaves from different plants and only considering second youngest leaves (fully expanded) per treatment on 21 August, according to [92]. Net photosynthesis (Pn), stomatal conductance to water vapor (gs), and transpiration (E) were obtained under photosynthetic steady-state conditions after ca. 2 h of illumination (in the middle of morning). A portable open-system infrared gas analyzer (Li-Cor 6400, Li-Cor, Lincoln, NE, USA) was used under environmental conditions, with external CO_2_ (ca. 400 ppm) and photosynthetic photon flux density (PPFD) ranging between 1200–1400 µmol m^−2^ s^−1^. Leaf instantaneous water use efficiency (iWUE) was calculated as the Pn-to-E ratio, indicating the units of assimilated CO_2_ per unit of water lost through transpiration.

### 4.5. Quantification of Zn Concentration in the Leaf

Zinc concentration was determined on 10 selected leaves from different plants and considering only the second youngest leaves (fully expanded) per treatment (i.e., after drying at 60 °C until a constant weighted, grounded, and processed into pellet) on two evaluation dates (samples harvest on 20 May and 24 July of 2019): without Zn foliar application and after the 4th Zn foliar application. Measurements were performed in triplicate, using an XRF analyzer (modelXL3t 950 He GOLDD+, Munich, Germany) under helium atmosphere and with emission of radiation for 180 s. For data analysis, NITON Data Transfer software (XL 3t-36653) was used [93].

### 4.6. Quantification of Zn Concentration in the Grapes at Harvest

At harvest, Zn concentration of randomized grapes was determined (i.e., after being washed, dried at 60 °C until constant weight, and grounded in an agate mortar). Then, an acid digestion procedure was performed with a mixture of HNO_3_^−^:HClO_4_ (4:1) [94], followed by filtration. Measurements were carried out in triplicate, using an atomic absorption spectrophotometer model, the Perkin Elmer Analyst 200 (Waltham, MA, USA), fitted with a deuterium background corrector and using the AA WinLab software program Version 32.

### 4.7. Morphometric and Colorimetric Parameters of Grapes at Harvest

At harvest, density, dry weight, and total soluble solids were measured considering three grapes randomly selected per treatment. Total soluble solids were measured in grape juice using a digital refractometer from Atago (Atago, Tokyo, Japan), and the values obtained were expressed as °Brix.

Colorimetric parameters were accessed at harvest, considering triplicates of three independent randomized grapes. The methodology followed [95], where brightness (L*) and chromaticity parameters (a* and b* coordinates) were obtained using a fixed wavelength, with Minolta CR 300 colorimeter (Minolta Corp., Ramsey, NJ, USA) coupled to a sample vessel (CR-A504). Using the illuminant D65, the system of the Commission Internationale d’Éclaire (CIE) was applied [95]. The parameter L* represented the brightness of the sample, with a range between 0 (black) and 100 (white). Parameters a* and b* indicate color variations between red (+60) and green (−60), and between yellow (+60) and blue (−60), respectively. The approximation of these coordinates to the null value is considered neutral colors such as white, gray, and black.

### 4.8. Statistical Analysis

Data were statistically analyzed using a one-way or two-way ANOVA (*p* ≤ 0.05) to determine differences between treatments and between both *Vitis vinifera* L. cv. Castelão and Syrah in the same treatment. After, a Tukey’s test for mean comparison was performed (all the tests with a 95% confidence level).

## 5. Conclusions

This study showed the efficiency of Zn biofortification with ZnSO_4_ and ZnO at concentrations of 900 and 1350 g ha^−1^ on Castelão and Syrah winegrapes. Indeed, both sources of Zn led to a higher accumulation of this nutrient, although the ZnO at the higher concentration was more efficient revealing a 4.3- and 2.3-fold increase in Castelão and Moscatel, respectively. Biofortification seems to be a strategy that allows a higher accumulation of Zn in grapes without a negative impact from a physiological perspective. Although, for a better understanding and process optimization, it is necessary more studies with other varieties and under different conditions and other Zn sources.

## Figures and Tables

**Table 1:** Average ± S.D. of normalized difference vegetation index (NDVI) of the vineyards of Castelão and Syrah with ZnO and ZnSO_4_ at different concentrations. Information collected after the 4th foliar application on 22 August 2019.

Treatments	Lagameças Field	Biscaia Field
Control	0.56 ± 0.16	0.66 ± 0.20
ZnO (900 g ha^−1^)	0.52 ± 0.18	0.60 ± 0.21
ZnO (1350 g ha^−1^)	0.53 ± 0.21	0.61 ± 0.20
ZnSO_4_ (900 g ha^−1^)	0.54 ± 0.17	0.65 ± 0.18
ZnSO_4_ (1350 g ha^−1^)	0.48 ± 0.17	0.55 ± 0.21

**Table 2:** Average ± S.E. of leaf gas exchange parameters, net photosynthesis (Pn), stomatal conductance to water vapor (gs), as well as variation in the instantaneous water use efficiency (iWUE = Pn/E) in leaves of *Vitis vinifera*, varieties Castelão and Syrah, after the 4th leaf spraying on 21 August with ZnO and ZnSO_4_ at different concentrations. For each parameter and variety, the average value ± S.E. (*n* = 6) is succeeded by different letters indicating significant differences between testing parameters for the different treatments (a, b) (statistical analysis using the single factor ANOVA test, *p* < 0.05).

Treatment	Pn (µmol CO_2_ m^−2^ s^−1^)	g_s_ (mmol H_2_O m^−2^ s^−1^)	E (mmol H_2_O m^−2^ s^−1^)	iWUE (mmol CO_2_ mol^−1^ H_2_O)
***Vitis vinífera* cv. Castelão**				
Control	5.8 ± 0.9 a	64.6 ± 16.3 a	2.0 ± 0.4 a	3.1 ± 0.2 a
ZnO (900 g ha^−1^)	6.7 ± 1.6 a	87.1 ± 32.8 a	2.6 ± 0.7 a	2.7 ± 0.2 a
ZnO (1350 g ha^−1^)	7.5 ± 0.8 a	84.3 ± 16.8 a	2.8 ± 0.4 a	2.8 ± 0.1 a
ZnSO_4_ (900 g ha^−1^)	4.6 ± 0.9 a	53.1 ± 9.2 a	2.0 ± 0.2 a	2.3 ± 0.2 a
ZnSO_4_ (1350 g ha^−1^)	3.9 ± 1.0 a	53.5 ± 12.7 a	2.0 ± 0.4 a	1.7 ± 0.2 a
***Vitis vinífera* cv. Syrah**				
Control	9.8 ± 0.4 b	251.8 ± 50.3 a	2.7 ± 0.2 b	3.7 ± 0.2 a
ZnO (900 g ha^−1^)	12.0 ± 1.2 ab	255.1 ± 57.7 a	3.2 ± 0.3 b	3.8 ± 0.1 a
ZnO (1350 g ha^−1^)	14.0 ± 0.8 a	267.3 ± 39.9 a	3.9 ± 0.2 ab	3.7 ± 0.1 a
ZnSO_4_ (900 g ha^−1^)	13.8 ± 0.5 a	213.8 ± 24.3 a	3.6 ± 0.2 ab	3.8 ± 0.1 a
ZnSO_4_ (1350 g ha^−1^)	13.3 ± 0.5 a	263.1 ± 17.6 a	4.3 ± 0.2 a	3.1 ± 0.1 a

**Table 3:** Average ± S.E. (*n* = 3) zinc concentrations in grapes at harvest of *Vitis vinifera,* varieties Castelão and Syrah. Letters a, b indicate significant differences among treatments in each variety, whereas letters A and B indicate the significant differences between each treatment of both varieties (statistical analysis using the two-way ANOVA test, *p* < 0.05).

Treatments	Zn (mg kg^−1^)	
	*Vitis vinífera* cv. Castelão	*Vitis vinífera* cv. Syrah
Control	1.5 ± 0.2 bA	4.5 ± 1.1 bA
ZnO (900 g ha^−1^)	2.4 ± 0.0 bB	7.4 ± 0.3 abA
ZnO (1350 g ha^−1^)	6.5 ± 0.5 aA	10.4 ± 1.5 aA
ZnSO_4_ (900 g ha^−1^)	2.4 ± 0.3 bB	6.1 ± 1.1 abA
ZnSO_4_ (1350 g ha^−1^)	5.7 ± 0.6 aB	8.3 ± 0.3 abA

**Table 4:** Average ± S.E. (*n* = 3) of dry weight, density, and total soluble solids (expressed as °Brix) in grapes of *Vitis vinifera,* varieties Castelão and Syrah. Letters a, b indicate significant differences could among treatments in each variety, whereas letters A and B indicate the significant differences between each parameter for both varieties in each treatment (statistical analysis using the two-way ANOVA test, *p* < 0.05).

Treatments	*Vitis Vinifera* L.					
	Castelão	Syrah	Castelão	Syrah	Castelão	Syrah
	Dry weight (%)		Density (g cm^−3^)		%TSS (°Brix)	
Control	27.6 ± 0.7 aA	25.1 ± 0.5 aA	1487 ± 40 aA	1049 ± 17 aB	26.3 ± 0.9 aA	18.7 ± 1.7 bB
ZnO (900 g ha^−1^)	27.9 ± 1.0 aA	24.0 ± 1.5 aA	1296 ± 45 aA	1056 ± 20 aB	22.0 ± 0.6 abA	19.3 ± 0.7 bB
ZnO (1350 g ha^−1^)	25.7 ± 1.0 aA	26.1 ± 0.9 aA	1274 ± 76 aA	1113 ± 58 aA	25.7 ± 1.8 aA	24.3 ± 0.3 aA
ZnSO_4_ (900 g ha^−1^)	24.7 ± 0.2 aA	24.4 ± 0.8 aA	1288 ± 86 aA	1143 ± 76 aA	20.8 ± 0.4 bA	19.7 ± 0.9 abA
ZnSO_4_ (1350 g ha^−1^)	25.1 ± 0.6 aA	26.4 ± 0.3 aA	1282 ± 136 aA	1108 ± 38 aA	22.0 ± 0.6 abA	21.0 ± 1.0 abA

**Table 5:** Average ± S.E (*n* = 3) of colorimeter parameters of the skin of grapes of *Vitis vinifera,* varieties Castelão and Syrah. Letter a indicates the absence of significant differences among treatments in each variety, whereas letters A and B indicate the significant differences between each parameter for both varieties in each treatment (statistical analysis using the two-way ANOVA test, *p* < 0.05). The L* parameter represents the brightness of the sample, with a range between 0 (black) and 100 (white). The parameters a* and b* indicate color variations between red (+60) and green (−60), and between yellow (+60) and blue (−60), respectively.

Treatments	Colorimeter Parameters					
	*Vitis vinífera* cv. Castelão			*Vitis vinífera* cv. Syrah		
	L	a *	b *	L	a *	b *
Control	21.6 ± 3.4 aA	−1.0 ± 0.3 aA	−1.9 ± 0.2 aB	18.3 ± 0.7 aA	0.3 ± 0.6 aA	−0.3 ± 0.4 aA
ZnO (900 g ha^−1^)	15.2 ± 1.4 aA	−2.2 ± 0.8 aA	3.1 ± 3.3 aA	21.0 ± 4.0 aA	0.3 ± 0.6 aA	0.3 ± 1.2 aA
ZnO (1350 g ha^−1^)	16.7 ± 1.1 aB	−1.4 ± 0.4 aB	0.2 ± 0.6 aA	27.1 ± 1.8 aA	0.1 ± 0.2 aA	−2.3 ± 0.1 aB
ZnSO_4_ (900 g ha^−1^)	18.8 ± 4.4 aA	−1.0 ± 0.4 aA	0.0 ± 2.1 aA	19.8 ± 2.9 aA	−0.9 ± 0.1 aA	−1.2 ± 0.5 aA
ZnSO_4_ (1350 g ha^−1^)	16.9 ± 2.9 aA	−1.3 ± 0.2 aB	0.0 ± 1.8 aA	16.2 ± 2.4 aA	−1.0 ± 0.3 aA	−0.6 ± 0.3 aB

## Data Availability

Not applicable.

## Abbreviations

Pn = net photosynthesis; gs = stomatal conductance to water vapor; E = transpiration; PPFD = photosynthetic photon flux density; iWUE = leaf instantaneous water use efficiency, calculated as the Pn-to-E ratio, indicating the units of assimilated CO_2_ per unit of water lost through transpiration.; °Brix = total soluble solids.
